# Sic Transit—Demise of the “Southern Medical Review”

**Published:** 1894-09

**Authors:** 


SIC TRANSIT.—It is not as easy to establish a medical journal in Texas as falling off of a log, but there be those—inexperienced persons—who seem to think it is. Our readers may remember having seen a copy—a single issue made its appearance—of the "*Southern Medical Review*." Texas was too small; it was going to spread out all over the South, and fill a long-felt want. Dr. Phenix was the name of the editor and proprietor, and the *Southern Medical Review* (for June) was issued from Houston.

The *Texas Sanitarian*, our neighbor and respected contemporary, kindly went to the relief of Dr. Phenix and took the thing off his hands, agreeing to carry out the few advertising contracts *The Review* had, and make good the fifty subscribers, mostly in Houston, it had already gotten. Dr. Phenix, we learned, said he had received a great deal of encouragement in his enterprise, but it was not convertable or negotiable, hence the early collapse of the *Southern Medical Review*. We had wished it better luck, though we were not surprised at its failure. It is not as easy as falling off a log to establish a good medical journal.

